# Depression and associated risk factors among hypertensive patients in primary health care centers in Dammam, Kingdom of Saudi Arabia

**DOI:** 10.11604/pamj.2021.38.278.27133

**Published:** 2021-03-17

**Authors:** Sumood Abdulbaqi Albasara, Mohammad Shafee Haneef, Mubashir Zafar, Khawaja Ghulam Moinuddin

**Affiliations:** 1 Department of Public Health, College of Public Health, Imamm Abdul Rehman Bin Faisal University, Dammam, Saudi Arabia,; 2 Department of Family and Community Medicine, College of Medicine, University of Hail, Hail, Saudi Arabia,; 3 National Guard Health Affairs, Riyadh, Saudi Arabia

**Keywords:** Depression, hypertension, income, medication, patient

## Abstract

**Introduction:**

depression is a common mental illness and Hypertension is a chronic disease and due to the negligence depression is co-morbidity with hypertension. The aim of this study was to determine the prevalence of depression and its associated factor among hypertensive patients in Dammam.

**Methods:**

it was a cross-sectional study and 342 hypertensive patients were selected from primary health care centers in Dammam, KSA. Beck Depression Inventory scale was used to determine the depression. The logistic regression analysis was used to assess the correlation between depression (dependent) and other variables. The P-value of less than 0.05 has been set to be statistically significant.

**Results:**

the proportion of depression with various degrees of severity among study participants was 19.6%. After Adjustment of covariates, older age [aOR 3.33, 95% CI 1.22-9.10; p= 0.019,low income [a OR 3.19, 95% CI 1.02-9.11; p= 0.003, illiterate [aOR 3.41, 95% CI 1.09-7.65; p= 0.005], physical inactivity [aOR 2.65, 95% CI 1.21-9.65; p= 0.051], ever smoker [aOR 2.99, 95% CI 1.09-3.77; p= 0.002], long standing hypertension > 3 years [aOR 3.765, 95% CI 1.01-7.10; p= 0.049] were more likely significantly associated with depression among hypertensive patients.

**Conclusion:**

depression is common among hypertensive patients in our setting. The associated multiple risk factors are older age, low income (< 3000 SR), physical inactivity, long standing hypertension.

## Introduction

Hypertension affects around 15% of the global population, which is amounting to over one billion people [1]. Hypertension is the blood pressure of the arteries elevates, as the systolic blood pressure starts to exceed 130-139 mmHg and the diastolic blood pressure exceeds 80-89mmHg [2]. Depression is a common mental illness and treatable disease, while typically is undiagnosed due to lack of awareness among physicians during treatment of patients having chronic diseases [3]. This typically leads to a negative impact on the patient´s mental health, which may affect their compliance with the chronic disease treatment and, in some cases, may lead to suicide attempts [4].

A systematic review and meta-analysis study which was conducted in China, (to assess the prevalence of depression in a patient known to have hypertension). This study showed that 26.8% of the population has depression with hypertensive [5]. Another study conducted in 2016 in Afghanistan on 234 hypertensive patients showed that 58.1% hypertensive patients were having depression and usually associated with age, diabetes mellitus, and two or more chronic diseases [6].

A study on 165 hypertensive patients had symptoms of depression, which represent 40% of the study subject with significant causes such as gender, age, socioeconomic status, the severity of hyper attention and other health behavior as exercise, healthy diet and smoking [7]. In India (Kanyakumari), a study took place that had similar findings of 40% with the depression symptoms associated with factors such as age, gender, family history of hypertension and other medical illness [8]. A study conducted in Malaysia, 32% was found the prevalence of depression in hypertensive patients [9]. A study was conducted and findings from the study showed that 24.7% maintained depression [10]. Another study, comparing the findings between Ghana and Nigeria, found the prevalence of depression among hypertensive patients were 41.7% in Ghana vs 26.6% in Nigeria [11]. In other words, considering depression in chronic disease is essential for public health in finance and improving life quality [4,12].

Few studies reached the prevalence of hypertensive patients suffering from this mental problem. Analysis of the Saudi population on this subject (especially in the Dammam area) has not been conducted to understand such disease, which affects the community. Furthermore, this study will also support widening the awareness of such disease, which will lead to general physicians´ ability to consider depression as one of the significant public health issues. This study aims to determine the prevalence of depression and its associate factors among hypertensive patients in Dammam, Saudi Arabia.

## Methods

**Study setting and study population:** study was conducted in the primary health care centers (PHCCs) in Dammam. Five PHCCs were included in the study, and these five PHCCs were selected by stratified random sampling according to the geographical division of Dammam (south (Al Jameen PHC which serving 3700), north (Al Azeeziah PHC which serving 7500 population), east (Al-Qadisiah PHC which serving 6500 community), west (Bader PHC which serving 10900 population) and centers (Qhurnatah PHC which serving 5400 population) these five PHCCs had the highest frequency of visitors for the chronic clinic.

**Study design:** community-based cross-sectional study, inclusion criteria: all patients attending the chronic clinic with confirmed diagnosis of hypertension, age group 20-80 years were included in the study. Exclusion criteria: any patient not will to participate, mentally ill patients has been excluded from participating in the study. The sample conducted for studying the prevalence of depression among hypertensive patients based on a previous study [13] at a proportion rate of 66%, at a significance level of 5% and confidence level of 95% would be 342 subjects. A uniform allocation was applied to select 69 participants from each of the five PHCCs. Participants were selected by convenience sampling after using the inclusion and exclusion criteria.

**Data collection:** data were obtained through self-administrated questionnaires distributed among hypertensive patients attending the PHCCs. There are two sections of questionnaire: First socio-demographic characteristics including age, gender, nationality, marital status, physical activity, educational level, employee status, smoking habit, duration of hypertension, hypertension control (by taking BP measurement by electronic sphygmomanometer before starting the interview), number of received antihypertensive medications, other medical condition and family history of hypertension or depression. The second section was the Beck Depression Inventory scale questionnaire (BDI ll) (Arabic version): This scale consists of 21 items self-reporting instrument to evaluate the severity of depression in a standard and psychiatric population. Each question was a 4-point scale range 0-3 in severity. The minimum score was zero, and the maximum was sixty-three. With this, the scoring system breakpoints were 0-13 standard, 14-19 mild depression, 20-28 moderate depression, and 29-63 severe depression [14].

**Statistical analysis:** the IBM SPSS Statistics version 23 was used for statistical analysis. The use of frequency and percentage achieved data description. The depression score was calculated by summation of the individual scores (inputted manually into the system). The binary logistic regression analysis was used to assess the association between depression (dependent) and other variables. Regarding binary logistic regression, the dependent variable (Depression) was dichotomous (No depression < 14 and depression >= 14). P-value of less than 0.05 was set to be statistically significant.

**Ethical consideration:** written informed consent was obtained from each participant, Ethical approval (IRB-PGS-2019-03-344) was obtained from Imam Abdurrahman Bin Feisal University's Research Ethical Review Board. All procedures performed in studies involving human participants were in accordance with the ethical standards of the institutional and/or national research committee and with the 1964 Helsinki declaration

## Results

**General Characteristics:** mean age of participants were 33 years with SD 1.35 years. Majority (55.8%) of participants were female, 61.7% of students were educated secondary level, 66.4% were un-employed, 63.4% were monthly income in the range of 3000-10000 SR. Majority (50.3%) of participants were doing physical activity (>150 minutes/week) and 66.4% were non-smoker (Table 1). Most (54.4%) of the participants were < 3 years of diagnosed hypertension, 57.6% were uncontrolled hypertension and 88.4% of participants were not taking any medication for hypertension (Table 2).

**Table 1:** socio-demographic characteristics of study participants (n=342)

Characteristics	Frequency (n)	Proportion (%)
**Age (Mean Age ± SD)**	33 ± 1.35	
20-50	152	44.45
51-70	190	55.55
**Gender**		
Male	151	44.2
Female	191	55.8
**Marital Status**		
Single	20	5.8
Married	246	71.9
Divorced	76	22.3
**Nationality**		
Saudi	269	78.7
Non-Saudi	6	1.8
**Education Level**		
Illiterate	86	25.1
Secondary education	211	61.7
University and higher	45	13.2
**Employed Status**		
Employed	115	33.6
Unemployed	227	66.4
**Monthly Income (SR)**		
<3000	79	23.1
3000-10000	217	63.4
>10000	46	13.5
**Physical Activity**		
< 150 minutes/ week	170	49.7
>150 minutes/ week	172	50.3
**Smoking Status**		
Non-smoker	227	66.4
Ever smoker	115	33.6
**Family history of depression**		
Yes	35	10.2
No	307	89.8

**Table 2:** hypertension characteristics of study participants (n=342)

Characteristics	Frequency (n)	%
**Duration of Diagnosed hypertension**		
< 3 years	186	54.4
≥ 3 years		
**Hypertension control**	156	45.6
Controlled	145	42.4
Uncontrolled		
**Family history of hypertension**	197	57.6
Yes	281	82.2
No	61	17.8
**Anti-Hypertensive Medication**		
Yes	74	21.6
No	268	88.4

**Prevalence of depression:** participants were suffering from depression as mild 4.97%, moderate 11.69%, severe 2.94% and total proportion of depression were 19.6% (Table 3).

**Table 3:** prevalence of depression among study participants (n=342)

Depression level	Frequency (n)	%
**Normal**	275	80.4
**Mild**	17	4.97
**Moderate**	40	11.69
**Severe**	10	2.94
**Total no of participant depression**	67	19.6

Correlates of hypertension: in univariate analysis, monthly income and family history of hypertension were statistically significant. After adjustment of covariates, older age [aOR 3.33, 95% CI 1.22-9.10; p= 0.019, low income [a OR 3.19, 95% CI 1.02-9.11; p= 0.003, illiterate [aOR 3.41, 95% CI 1.09-7.65; p= 0.005], physical inactivity [aOR 2.65, 95% CI 1.21-9.65; p= 0.051], ever smoker [aOR 2.99, 95% CI 1.09-3.77; p= 0.002], long standing hypertension > 3 years [aOR 3.765, 95% CI 1.01-7.10; p= 0.049] were more likely significantly associated with depression among hypertensive patients (Table 4).

**Table 4:** socio-demographic and hypertension factors associated with depression among study participants

Characteristics	Depression		Depression	
	Crude Odd Ratio (Confidence Interval)	P-value	Adjusted Odd Ratio (Confidence Interval)	P-value
**Age**				
20-50	1		1	0.019
51-70	1.347(0.98-8.98)	0.081	3.33(1.22-9.10)	
**Gender**				
Female	1	0.12	1	0.091
Male	0.98(0.02-4.76)		2.43(0.99-6.7)	
**Monthly Income (SR)**				
>10000	1	0.004	1	0.003
3000-10000	6.29(1.34-11.23)		6.28(2.36-9.38)	
<3000	1.42(0.87-8.98)		3.19 (1.02-9.11)	
**Education Level**				
Secondary Education	1		1	0.005
Primary education	1.76(0.92-5.34)	0.092	2.01(1.23-5.98)	
Illiterate	1.98(1.01-6.65)		3.41(1.09-7.65)	
**Employed Status**				
Employed	1	0.112	1	0.002
Unemployed	0.99(0.34-4.87)		1.39(1.10-7.87)	
**Physical Activity**				
>150 minutes/ week	1		1	
< 150 minutes/ week	0.34(0.10-2.54)	0.13	2.65(1.21-9.65)	0.051
**Smoking Status**				
Non-smoker	1	0.079	1	0.002
Ever smoker	1.43(0.54-5.76)		2.99(1.09-3.77)	
**Duration of Diagnosed hypertension**				
< 3 years	1		1	0.049
≥ 3 years	2.651(1.98-8.98)	0.051	3.765(1.01-7.10)	
**Hypertension control**				
Controlled	1		1	
Uncontrolled	0.76(0.10-3.23)	0.143	0.89(0.34-4.54)	0.089
**Family history of hypertension**				
No	1	0.024	1	0.013
Yes	0.29(0.04-1.23)		1.28(0.36-9.38)	
**Anti-Hypertensive Medication**				
Yes	1		1	
No	1.26(0.82-3.34)	0.092	2.11(1.01-8.23)	

## Discussion

As a first study discussing the prevalence of depression in hypertensive patients in the Eastern Province of Saudi Arabia, the result of our study showed that around 19.6% of the study subject had depression,4.97% as mild, 11.69% moderate, and severe cases reported at 2.94 %. There are different predictors which contributed to depression among hypertensive patients.

Results of this study found that age group between 51-60 years were suffering more depression. This result was contrast to previous study, cross-sectional find out about in Netherland [15] has said the decreasing trend of depression with the increase in age. From our discussions with the participants, we saw that this age group had the highest concerning thoughts about their dependents (children and direct family) in terms of well-being and life comfort. Study result found that physical activity is also contributor of depression among hypertension patients, those having less physical active were depressed. Which is similar to the population based prospective cohort study conducted in Washington [16] secondary data analysis from cross-sectional survey in Austria [17] and cross-sectional study in France [18].

Another risk, which was shown in the study, included the monthly income. With this in mind, participants that had a monthly income between 3,000 and 5,000 had the highest probability of becoming depressed. This is mainly because this monthly income segment has personal skills in developing their abilities. Similar studies have been conducted in neighboring areas, such as Jeddah and Bahrain, which showed similar findings of around 20% chance of becoming depressed within hypertensive patients (vs. our finding of 19.6% in our study that focuses on Dammam) [19,20]. Additional factors affecting the well-being included the duration of hypertension, as longer the illness, the higher probability of becoming depressed. This is normal, as the longer a patient is ill with the same disease, the less hope he has in a full recovery. Previous studies found that which is similar to the cross-sectional study conducted in Nepal [21].

Study result found that those patients not taking any drug were more likely associated with depression. Previous study found that those patients taking medicine and had controlled hypertension less likely associated with depression [22]. There are several limitations of this study. First it was the cross sectional study which did not determined the temporal relationship between outcome and independent variable. Second the cultural restrictions affected the ending findings of the BDI II scale, as the study subject hesitated in providing transparent answers for some of the questions.

## Conclusion

Finding of study shows that one third of participants were suffering from depression. Our study also shows that hypertensive patients are vulnerable to depression, involving multiple risk factors, such as age group, monthly income, medical illness, duration of hypertension, and several antihypertensive medications used. It is essential to educating the patients in an early stage (less than 50 years old) on the importance of medication, lifestyle, and future expectations.

### What is known about this topic

- Hypertension is a chronic disease and depression is common problem among chronic disease;
- Different studies were conducted to determine the association between hypertension and depression;
- These studies cover all populations including expatriate which did not determine the actual problem among Saudi women.

### What this study adds

- This study shows a prevalence of depression of 19.6%$ among patients with hypertension from primary health centers at Saudi Arabia;
- Factors associated with depression included: older age, illiterate, physical inactivity, ever smoker, long standing hypertension;
- The study results found a positive relationship between depression and hypertension.
